# LncRNAs specifically overexpressed in endocervical adenocarcinoma are associated with an unfavorable recurrence prognosis and the immune response

**DOI:** 10.7717/peerj.12116

**Published:** 2021-09-21

**Authors:** Yong Song, Long Nie, Yu-Ting Zhang

**Affiliations:** 1 Department of Public Health, Maternal and Child Health Hospital of Hubei Province, Tongji Medical College, Huazhong University of Science and Technology, Wuhan, Hubei, China; 2 School of Health Sciences, Wuhan University, Wuhan, Hubei, China; 3 Department of Oncology, Suizhou Hospital, Hubei University of Medicine, Suizhou, Hubei, China; 4 School of Nursing, Health Science Center, Shenzhen University, Shenzhen, Guangdong, China

**Keywords:** Cervical cancer, Endocervical adenocarcinoma, Long noncoding RNA, Recurrence, Immune response

## Abstract

**Background:**

Cervical cancer is the fourth most common gynecological tumor in terms of both the incidence and mortality of females worldwide. Cervical squamous cell carcinoma (CSCC) accounts for 70–80% of cervical cancers, and endocervical adenocarcinoma (EAC) accounts for 20–25%. Unlike CSCC, EAC has worse clinical outcomes and prognosis. In this study, we explored the relationship between various types of long noncoding RNAs (lncRNAs) and pathological types of cervical cancer.

**Methods:**

RNA sequencing (RNA-Seq) and clinical data from The Cancer Genome Atlas (TCGA) were used in this study. A single-sample gene set enrichment analysis (ssGSEA) and the ESTIMATE package were used to assess lncRNA activity and immune responses, respectively. RT-qPCR was performed to verify our findings.

**Results:**

We explored the relationship between various types of lncRNAs and pathological types of cervical cancer. A series of long intergenic noncoding RNAs (lincRNAs) and antisense RNAs, which are the major types of lncRNAs, were identified to be specifically expressed in EAC and associated with a poor recurrence prognosis in patients with cervical cancer, suggesting that they might serve as independent prognostic markers of recurrence in patients with cervical cancer. RT-qPCR was performed to verify the 10 EAC-specific lncRNAs in cervical cancer samples we collected. Furthermore, the overexpression of these lncRNAs was positively correlated with EAC pathology levels but negatively correlated with immune responses in the microenvironment of cervical cancer.

**Conclusions:**

These lncRNAs potentially represent new biomarkers for the prediction of the recurrence prognosis and help obtain deeper insights into potential immunotherapeutic approaches for treating cervical cancer.

## Introduction

Cervical cancer is the fourth most common gynecological tumor in terms of both the incidence and mortality of females worldwide, with an estimated 570,000 cases and 311,000 deaths occurring in 2018 (Bray et al., 2018; Vu et al., 2018). Cervical squamous cell carcinoma (CSCC) accounts for 70–80% of cervical cancers, and endocervical adenocarcinoma (EAC) accounts for 20–25% (Tsikouras et al., 2016). Unlike CSCC, the differential diagnosis of early invasive adenocarcinoma from EAC *in situ* to show the relatively complex architecture can be difficult to achieve (Ronnett, 2016). EAC has worse clinical outcomes and prognoses, although some studies have reported no differences in survival between patients with EAC and CSCC (Marth et al., 2017). Although EAC accounts for a lower proportion of cervical cancer cases, it causes a disproportionate number of deaths because of its aggressive behavior. Accordingly, the pathological types of cervical cancer and strategies to improve the prognosis of patients with EAC should be identified.

In humans, protein-coding genes account for less than 2% of the total genome. However, 70% of the human genome is transcribed into RNA, generating thousands of noncoding RNAs (Yan et al., 2015). The broad term long noncoding RNAs (lncRNAs) refers to transcripts >200 nt in length that do not contain a protein-coding sequence (Mattick & Rinn, 2015). Based on the genome position, lncRNA genes are further grouped into subclasses, including long intergenic noncoding RNAs (lincRNAs), natural antisense RNA transcripts, sense intronic RNAs, sense overlapping RNAs and processed transcripts (Jadaliha et al., 2018). LincRNAs are considered the largest class of lncRNA molecules. LincRNAs retain exon-intron structures and are located in highly regulated regions of the genome (Talyan, Andrade-Navarro & Muro, 2018). Natural antisense transcripts have been defined as overlapping antisense noncoding RNAs that regulate the expression of their opposite coding gene (Ounzain et al., 2015). Prior studies revealed tumor-suppressive or tumor-promoting roles for lncRNAs. For instance, lncRNAs correlated with cervical cancer include TERRA, HOTAIR, H19 and PVT1 (Bhan, Soleimani & Mandal, 2017; Gao et al., 2017; Guo et al., 2019; Oh et al., 2017; Ou et al., 2018). LncRNAs serve as vital regulators of gene expression in the immune response. According to a previous study, lncRNAs control the differentiation and function of innate and adaptive cell types (*e.g*., T cells and CD8 T cells) (Chen, Satpathy & Chang, 2017). To the best of our knowledge, no studies have reported the relationship among lncRNAs, different cervical cancer pathological types and immune responses.

Here, RNA sequencing (RNA-Seq) data from The Cancer Genome Atlas (TCGA) were employed to explore the association between various types of lncRNAs and the pathological types of cervical cancer. A group of lncRNAs, including lincRNAs and antisense RNAs, specifically overexpressed in EAC was identified. Furthermore, clinical data from TCGA were used to analyze the survival and recurrence of these lncRNAs. Finally, lncRNA overexpression was shown to be associated with immune responses in the cervical cancer microenvironment, particularly in EAC. This study may provide new prognostic markers and explain the mechanisms of lncRNA function in cervical cancer.

## Materials and Methods

### Raw data

RNA-Seq and clinical data from TCGA were downloaded from https://portal.gdc.cancer.gov/. The RNA-Seq data were reported as fragments per kilobase million (FPKM) (Cui et al., 2020; Gibb et al., 2015; Tang et al., 2021). The genotype classification was based on the Ensemble dataset (http://asia.ensembl.org/index.html).

### Definitions of clinical survival and recurrence types

Four clinical survival and recurrence types were selected here, namely, overall survival (OS), progression-free survival (PFS), disease-free survival (DFS) and disease-specific survival (DSS). These categories were defined as follows: OS was defined as the period from the date of diagnosis until death from any cause, DSS was the period from the date of the initial diagnosis until the date of last contact or death from another cause, PFS was the period from the date of diagnosis until the date of the first occurrence of a new tumor event, and DFS was the PFS of patients after their initial diagnosis and treatment (Liu et al., 2018; Wang, Yan & Ye, 2020).

### LncRNA score, tumor purity and immune score

A single-sample gene set enrichment analysis (ssGSEA) was conducted to assess the lncRNA (lincRNA and antisense RNA) scores based on expression (Hanzelmann, Castelo & Guinney, 2013). The ESTIMATE package was used to assess tumor purity and the immune score (Yoshihara et al., 2013). All these analyses were conducted using R software 4.0.3.

### Identification of target genes and enrichment analysis

Gene set enrichment analyses (GSEAs) were performed to identify the extent of the enrichment of the immune response in cervical cancer based on fold changes (FCs) in lncRNA expression (Long et al., 2019). Genes were arranged from large to small based on their FCs compared with the high and low expression groups. Subsequently, genes and FC values were listed and a gene ontology (GO) enrichment analysis was performed with GSEA in clusterProfiler (Yu et al., 2012). Target genes of lncRNAs were identified in ENCORI (http://starbase.sysu.edu.cn/index.php). Enrichment analysis of target genes were performed by Metascape (https://metascape.org/).

### Analyses of populations and subpopulations of infiltrating immune cells

T cell, B cell, CD8 T cell and NK cell molecular markers were built by a research group using genes specific for these immune cells (Bindea et al., 2013). T cell, B cell, CD8 T cell and NK molecular markers are as follows in Table S1. All these analyses were conducted using R software 4.0.3. Besides, TIMER and EPIC immune infiltrating data were downloaded from http://timer.cistrome.org/.

### RT-qPCR

Twelve groups of cervical cancer tissues and normal tissues were obtained from 12 patients who underwent radical resection at Suizhou Hospital, Hubei University of Medicine between June 2020 and September 2020. The clinical cases information was showed in Table S2. The expression levels of lncRNAs were examined using RT-qPCR. Total RNA was extracted by trozil, RNA was converted into cDNA using cDNA Synthesis Kit (TaKaRa, Shiga, Japan). Quantification of the cDNA template was performed with RT-qPCR using SYBR green as a fluorophore. All experimental procedures were approved by the Ethics Committee of Suizhou Hospital, Hubei University of Medicine. Written informed consent was provided by all patients prior to the study.

### Statistical analysis

The Mann–Whitney test was performed to assess the significance of differences in expression level between any two types of samples. The overlapping analysis was conducted using Venny 2.1 (http://bioinfogp.cnb.csic.es/tools/venny/index.html). Other analyses and plots were generated with GraphPad Prism 9 or R software 4.0.3.

## Results

### LincRNAs and antisense RNAs specifically expressed in EAC rather than other types of LncRNAs

**Figure 1:**
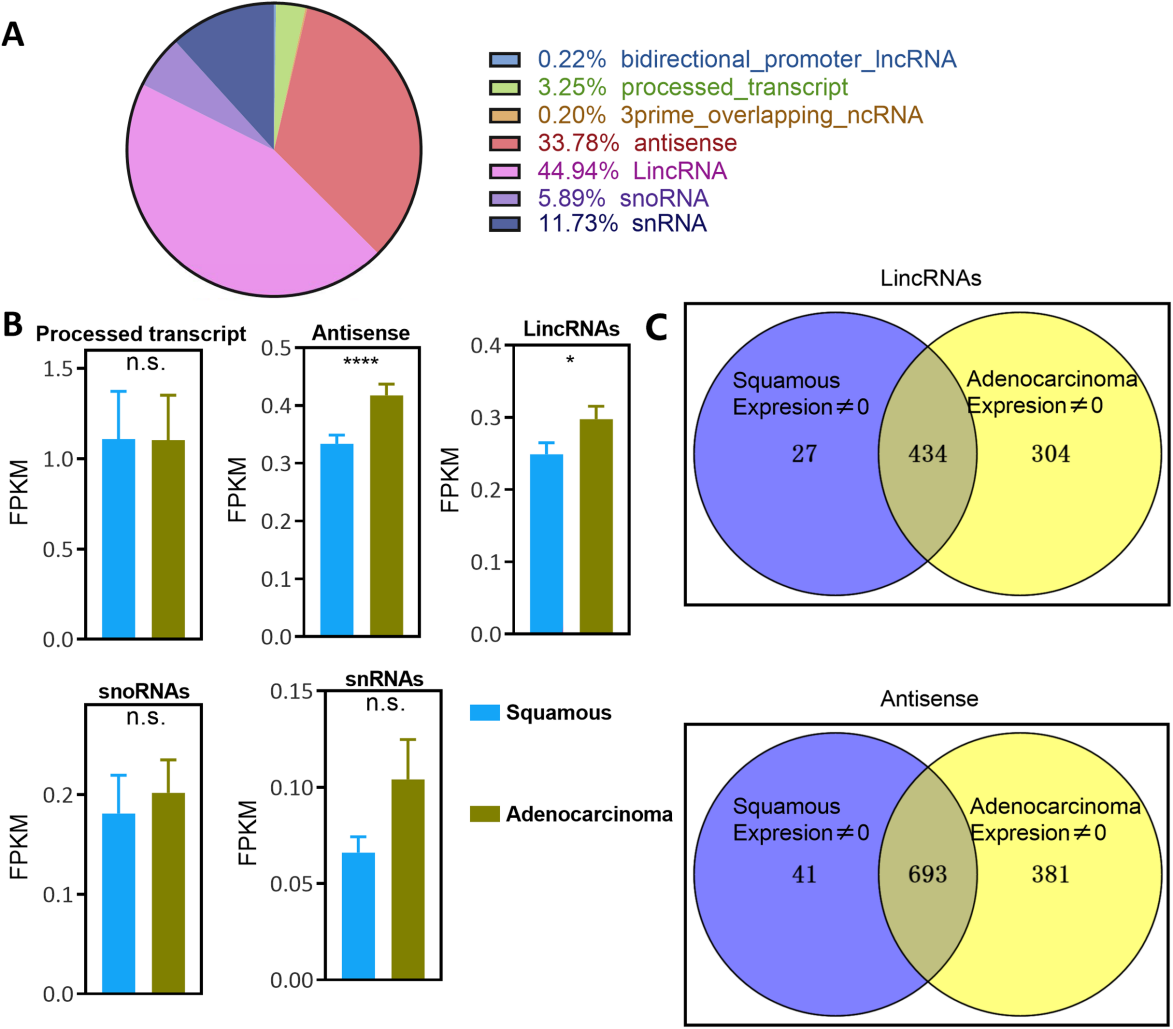
Landscape of LncRNAs expression in cervical cancer. (A) Pie plot of types of LncRNAs. (B) Bar plot vary of LncRNAs expression level in different types of pathology in cervical cancer. Statistics originate from Mann–Whitney test. *P < 0.05, ****P < 0.0001. (C) Venn plot of the LincRNAs and antisense whose expression level was not zero in EAC and CSCC samples in cervical cancer.

The expression of lncRNAs was profiled in a cohort of 299 cervical cancer samples (including 252 CSCC and 47 EAC samples) from TCGA datasets to identify lncRNAs that are dysregulated in the pathology of cervical cancer. Notably, lincRNAs and antisense RNAs, namely, the main types of lncRNAs, were significantly overexpressed in EAC samples compared with CSCC samples (Figs. 1A and 1B). However, processed transcripts, snoRNAs and snRNAs were not significantly differentially expressed in any type of CSCC or EAC sample (Fig. 1B). Thus, lincRNAs and antisense RNAs, but not other types of lncRNAs, were specifically expressed in serous EAC.

Numerous lncRNAs are expressed at very low levels or are not detected in cancer samples. The number of EAC and CSCC samples whose expression of lncRNAs was not zero was counted to reduce background noise and detect specific lincRNAs and antisense RNAs in EAC (Fig. 1C and Table S3). The expression of 304 lincRNAs and 381 antisense RNAs was nonzero only in EAC samples. These upregulated lncRNAs were defined as EAC lncRNAs. Twenty-seven lincRNAs and 41 antisense RNAs were not zero only in CSCC samples, and these lncRNAs were defined as CSCC lncRNAs. The expression of 343 lincRNAs and 693 antisense RNAs was not zero in both EAC and CSCC samples.

### EAC LncRNAs were differentially expressed in EAC and CSCC

**Figure 2:**
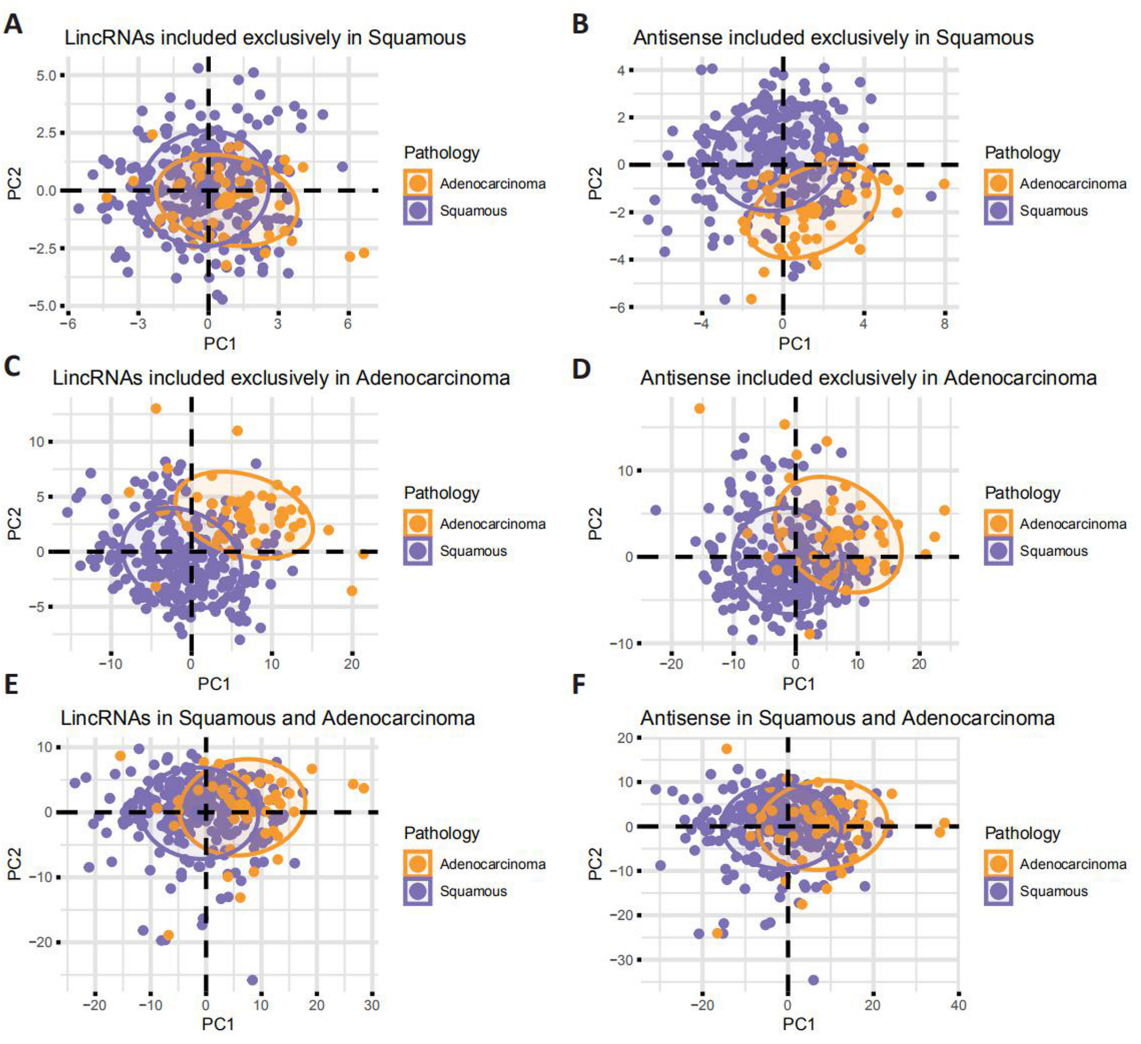
Landscape of LncRNAs differentially expressed between EAC and CSCC. (A–F) PCA plot showing the difference in expression of LncRNAs between EAC and CSCC samples in cervical cancer.

According to the principal component analysis (PCA), the correlation between EAC and CSCC samples was verified based on the expression of lincRNAs and antisense RNAs (Fig. 2). EAC samples were separate from CSCC samples in terms of the expression of EAC lncRNAs (Figs. 2C and 2D), especially lincRNAs. EAC and CSCC samples were clustered together in terms of the lncRNAs that were expressed in both EAC and CSCC samples (Figs. 2E and 2F). For CSCC lncRNAs, EAC samples were separate from CSCC samples in terms of the expression of antisense RNAs were clustered together in the expression of lincRNAs (Figs. 2A and 2B). Based on these results, EAC lncRNAs and antisense RNAs were differentially expressed in EAC and CSCC.

### EAC LncRNAs were significantly overexpressed in EAC but not CSCC

**Figure 3:**
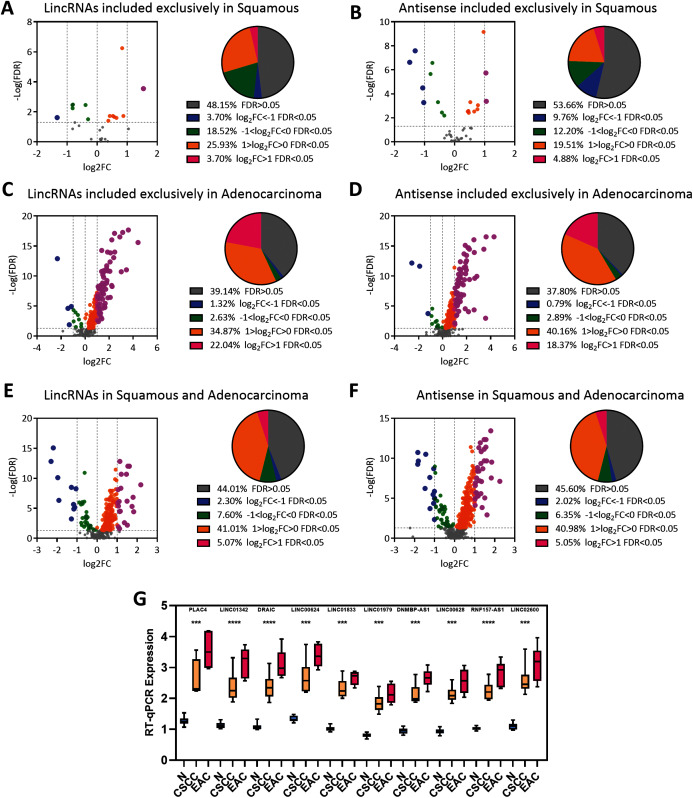
EAC LncRNAs were significantly overexpressed in EAC. (A–F) Volcano plot showing the significant dysregulation of lncRNAs. Pie plot of lncRNAs dysregulated to different extents. Statistical analyses were performed using the Mann–Whitney U test. (G) Boxplot showing the expression of ten lncRNAs in normal, CSCC and EAC samples determined using RT-qPCR. Statistical analyses were performed using the Kruskal–Wallis test.

A differential expression analysis was conducted using EAC and CSCC samples to clarify the dysregulation of these lncRNAs in the pathology of cervical cancer. The analysis here revealed that most CSCC lncRNAs were not dysregulated in any pathological type of cervical cancer (lincRNAs: 48.15%; antisense RNAs: 53.66%), and half of the remaining lncRNAs were upregulated in EAC and CSCC (Figs. 3A and 3B). Interestingly, most EAC lncRNAs were significantly upregulated in EAC (lincRNAs: 60.86%; antisense RNA: 62.20%) (Figs. 3C and 3D). These upregulated lncRNAs were defined as lncRNAs specifically expressed in EAC. Although lncRNAs that were expressed in both EAC and CSCC samples were upregulated in EAC (lincRNAs: 55.99%; antisense RNA: 54.40%), the proportion of the group in which the FC > 2 was much lower in CSCC lncRNAs than in EAC lncRNAs (lincRNAs: 5.07%: 22.04%, antisense RNA: 5.05%: 18.37%) (Figs. 3E and 3F).

We randomly chose 10 lncRNA candidates with an FC > 1 (Table 1) and validated their expression in 12 paired cervical cancer samples, including 12 normal, 6 CSCC and 6 EAC samples, we collected from the hospital to verify the expression of the EAC lncRNAs identified above. RT-PCR revealed that these lncRNAs were upregulated in cervical cancer samples, particularly in EAC samples (Fig. 3G). Thus, EAC lncRNAs were significantly overexpressed in EAC but not in CSCC.

**Table 1:** Information of selected genes.

Gene stable ID	Gene name	Gene type
ENSG00000280109.1	PLAC4	Antisense
ENSG00000223823.1	LINC01342	LincRNA
ENSG00000245750.6	DRAIC	LincRNA
ENSG00000278811.3	LINC00624	Antisense
ENSG00000259439.2	LINC01833	LincRNA
ENSG00000262585.1	LINC01979	LincRNA
ENSG00000227695.4	DNMBP-AS1	Antisense
ENSG00000280924.1	LINC00628	LincRNA
ENSG00000267128.1	RNF157-AS1	Antisense
ENSG00000250986.1	LINC02600	LincRNA

### Overexpression of LncRNAs specifically expressed in EAC was associated with an unfavorable recurrence prognosis

**Figure 4:**
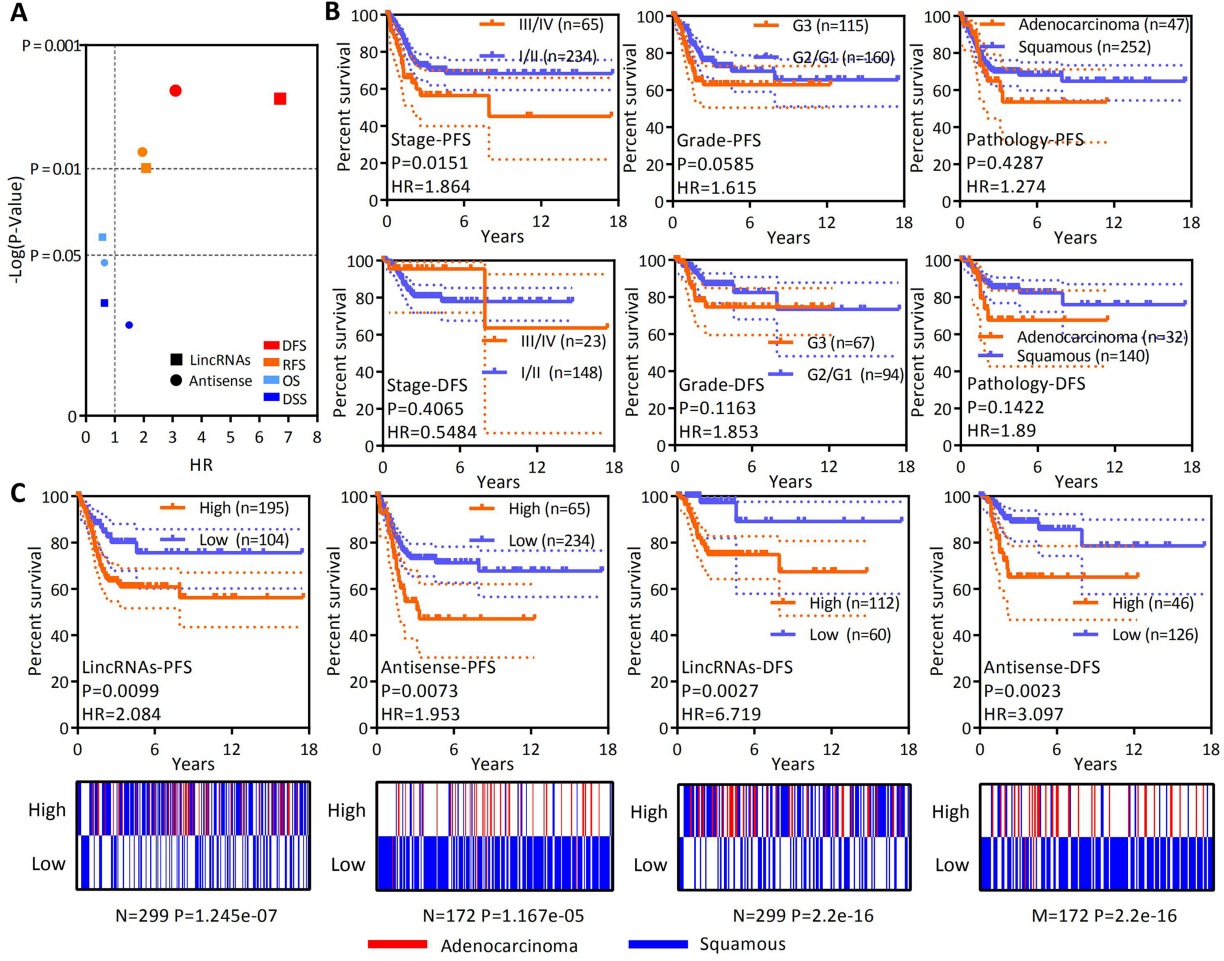
LncRNAs specifically expressed in EAC might serve as independent factors predicting the recurrence prognosis. (A) Dot plot showing the OS, DSS, DFS and PFS based on the expression of lncRNAs specifically expressed in EAC (lincRNAs and antisense RNAs) in patients with cervical cancer. (B) Kaplan–Meier plot showing the DFS and PFS of patients with cervical cancer stratified by the clinical stage, histological grade and pathological type. (C) Top panel: Kaplan–Meier plot showing the DFS and PFS of lncRNAs specifically expressed in EAC in patients with cervical cancer. Bottom panel: Heatmap showing the rates of different types of pathology (EAC and CSCC) among patients with cervical cancer.

OS is considered a vital endpoint, yet the exclusive use of OS as an endpoint may weaken a clinical study as deaths may occur. When OS and DSS are used, longer follow-up will be required. Thus, DFS and PFS are used in many clinical trials and serve as composites of tumor progression and death (Johnson, Liauw & Lassere, 2015; Punt et al., 2007). OS and DSS are associated with survival, and DFS and PFS are associated with recurrence. OS, DSS, DFS and PFS analyses were conducted to test whether the expression of lncRNAs specifically expressed in EAC represents a group to predict patient survival and the recurrence of cervical cancer. A ssGSEA was performed to assess the expression of all lncRNAs specifically expressed in EAC. The Z-score-transformed ssGSEA value served as the lincRNA score or antisense RNA score. Our analysis revealed that high lincRNAs and antisense RNA expression were associated with a poor recurrence prognosis rather than shorter survival (Fig. 4A). DFS and PFS analyses were conducted in patients stratified by clinical stage, histological grade and pathological type to investigate whether the levels of lincRNAs and antisense RNAs specifically expressed in EAC are independent factors predicting recurrence in patients with cervical cancer (Figs. 4B and 4C). The clinical stage (PFS: HR = 1.864, *P* = 0.0151; DFS: HR = 0.5484, *P* = 0.4065), histological grade (PFS: HR = 1.615, *P* = 0.0585; DFS: HR = 1.853, *P* = 0.1163) and pathological type (PFS: HR = 1.272, *P* = 0.4287; DFS: HR = 1.89, *P* = 0.1422) were not significantly or *P* value more than lincRNAs (PFS: HR = 2.084, *P* = 0.0099; DFS: HR = 6.719, *P* = 0.0027) or antisense RNAs (PFS: HR = 1.953, *P* = 0.0073; DFS: HR = 3.097, *P* = 0.0023). Furthermore, the levels of lincRNAs (*P* = 0.021) or antisense RNAs (*P* = 0.003) specifically expressed in EAC are still significantly associated with poor PFS in patients with cervical cancer according to Multivariate Cox regression analysis (Table 2). Based on these results, the expression levels of lincRNAs and antisense RNAs that are specifically expressed in EAC potentially represent independent prognostic factors for recurrence in patients with cervical cancer.

**Table 2:** Multivariate Cox regression analysis of PFS based on lincRNAs/antisense RNAs, stage, grade and pathology types.

Multivariate Cox	Groups	*P*	HR	95% CI
LincRNAs	High *vs*. low	0.021	2.038	[1.112–3.736]
Stage	III/IV *vs*. I/II	0.006	2.072	[1.230–3.490]
Grade	G3 *vs*. G1/G2	0.302	1.299	[0.791–2.133]
Pathology	Adenocarcinoma *vs*. squamous	0.607	1.184	[0.621–2.258]
**Multivariate Cox**	**Groups**	***P***	**HR**	**95% CI**
Antisense RNAs	High *vs*. low	0.003	2.787	[1.408–5.515]
Stage	III/IV *vs*. I/II	0.009	1.998	[1.188–3.362]
Grade	G3 *vs*. G1/G2	0.257	1.335	[0.810–2.199]
Pathology	Adenocarcinoma *vs.* squamous	0.295	0.639	[0.276–1.478]

### LncRNAs specifically expressed in EAC are involved in the immune response and cancer-related pathway

**Figure 5:**
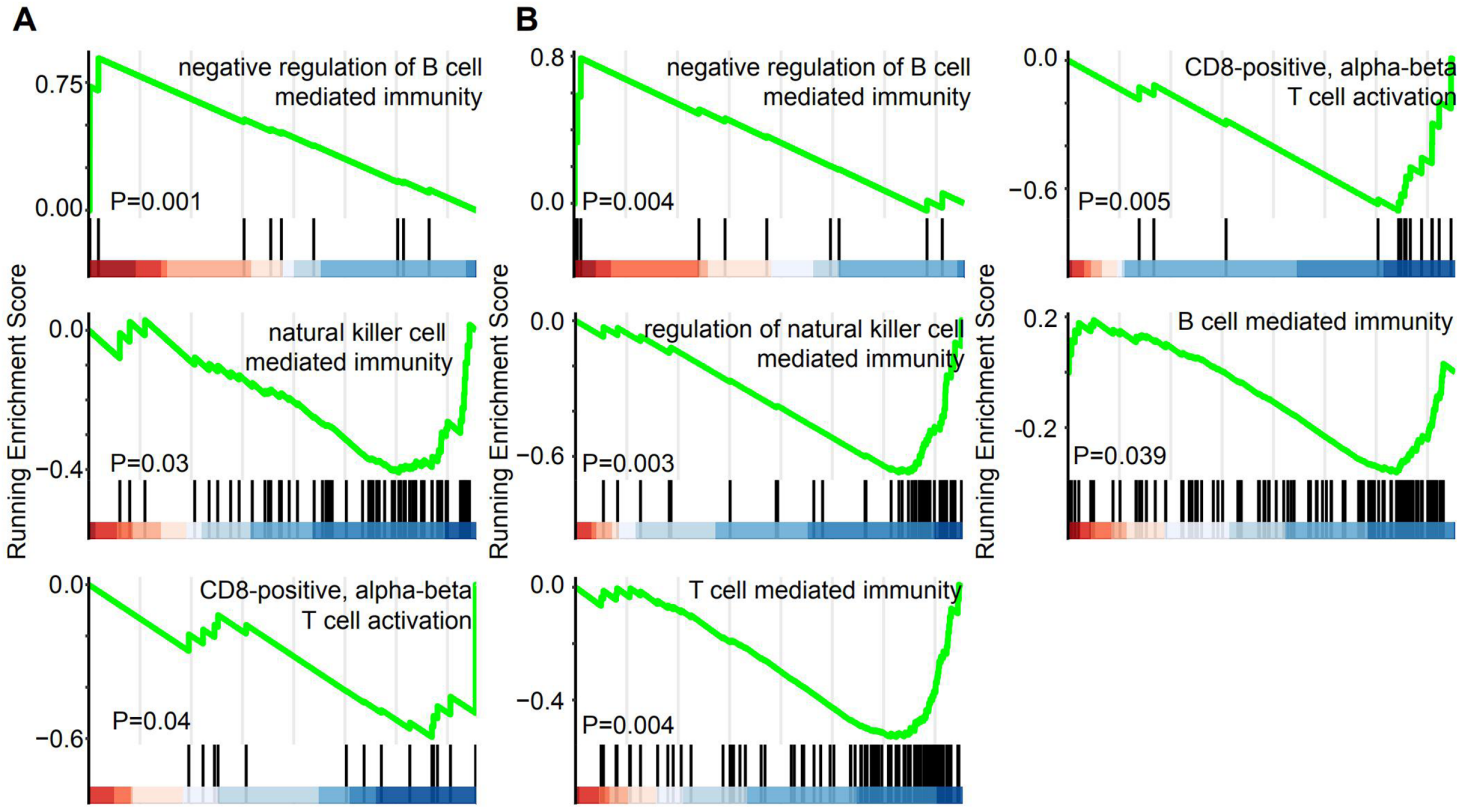
EAC Specifically expressed LncRNAs are involved in immune response. (A, B) GSEA demonstrating the enrichment of immune cells infiltration signature in the ranked gene list of EAC specifically expressed LincRNAs (A) and antisense (B) fold change with high and low group.

GSEA was performed in the GO term database to identify whether immune pathways correlated with the lncRNAs that were specifically overexpressed in EAC. Overexpression of lincRNAs specifically in EAC resulted in the enrichment of gene sets related to negative regulation of B cell-mediated immunity. Conversely, negative enrichment of natural killer cell-mediated immunity and CD8-positive alpha-beta T cell activation was observed after lincRNA overexpression (Fig. 5A). Similarly, genes involved in these three immune responses were enriched or negatively enriched in cervical cancer samples with overexpression of EAC-specific antisense RNAs (Fig. 5B). Furthermore, genes involved in T cell-mediated immunity and B cell-mediated immunity were negatively enriched in cervical cancer samples overexpressing EAC-specific antisense RNAs. Therefore, EAC-specific lncRNA overexpression may affect immune cell infiltration in cervical cancer. Furthermore, we selected top 10 EAC-specific lincRNA and antisense RNAs to analyze their target mRNA in ENCORI dataset. A total of six of them were matched with ENCORI dataset and 83 target genes were identified (Fig. S1). These target genes were dysregulated in multiple cancer types and associated with cancer-related pathway, such as “KEGG pathway in cancer” and “Reactome signaling by TGFB family members”. These results suggest that EAC-specific lncRNAs were associated with cancer-related pathway.

### EAC-specific LncRNA overexpression inhibits immune responses in the cervical cancer microenvironment, particularly in EAC

**Figure 6:**
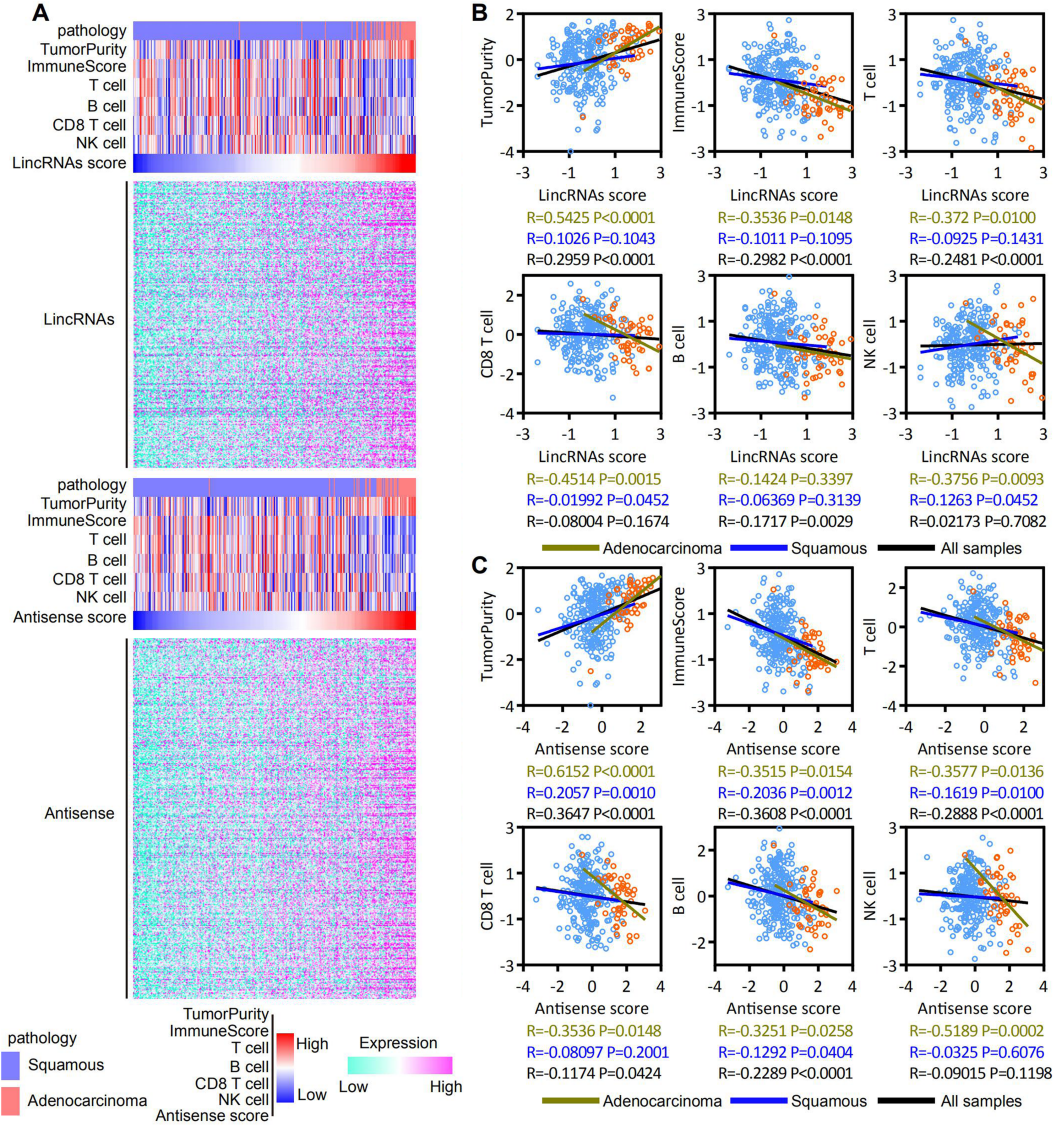
EAC specifically expressed LncRNAs overexpression inhibit the immune responses in the cervical cancer microenvironment, particularly in EAC. (A) Heatmap showing the correlation among expression of EAC specifically expressed LncRNAs (up: LincRNAs, bottom: antisense), pathology types, tumor purity, immune score, T cell, B cell, CD8 T cell and NK cell molecular markers activity. (B, C) Dot plot showing the correlation between LncRNAs (B: LincRNAs, C: antisense) and tumor purity, immune score, T cell, B cell, CD8 T cell and NK cell molecular markers activity, respectively.

ESTIMATE and ssGSEA were used to assess the tumor purity, immune score, and T cell, B cell, CD8 T cell and NK cell molecular marker activities and to identify immune cells that infiltrated into the cervical cancer microenvironment of patients with EAC-specific lncRNA overexpression. Our analysis revealed that lincRNA expression and tumor purity increased with an increasing lincRNA score. The EAC pathological type was associated with a high lincRNAs score. In contrast, the immune score and T cell, B cell, CD8 T cell and NK cell molecular marker activities decreased with an increasing lincRNA score (Fig. 6A, top panel). Antisense RNAs produced similar results to lincRNAs (Fig. 6A, bottom panel). A correlation analysis was conducted based on the lincRNA score, antisense RNA score, and immune score, and T cell, B cell, CD8 T cell and NK cell molecular marker activities to further determine the correlation between immune cell infiltration and the expression of EAC-specific lncRNAs (Figs. 6B and 6C). We also analyzed in robust methods, such as TIMER, EPIC and CIBERSORT (Fig. S2). Our analysis revealed that tumor purity was positively correlated with the lincRNAs score and antisense RNA score, particularly in EAC samples. The immune score and T cell and B cell molecular marker activities were negatively correlated with the lincRNA score and antisense RNA score, particularly in EAC samples. The activity of CD8 T cell and NK cell molecular markers was negatively correlated with the lincRNA score and antisense RNA score only in EAC samples. Notably, the correlation in CSCC samples was not significant. Based on these findings, the overexpression of lncRNAs specifically expressed in patients with EAC affected immune cell infiltration in the cervical cancer microenvironment, particularly in EAC.

## Discussion

Cervical cancer is now the fourth most common cancer in females worldwide. EAC, a pathological type of cervical cancer, has caused many deaths because of its aggressive behavior (Bray et al., 2018; Marth et al., 2017). Early studies revealed that lncRNAs were correlated with cervical cancer (Bhan, Soleimani & Mandal, 2017). The present study is the first to identify the expression levels of various types of lncRNAs in different pathological types of cervical cancer. LincRNAs and antisense RNAs were overexpressed in EAC, but not in other types (Fig. 1B), and lincRNAs and antisense RNAs accounted for the majority of the lncRNAs (44.94% and 33.78%, respectively) (Fig. 1A). Thus, lincRNAs and antisense RNAs are specific types of lncRNAs correlated with EAC. Next, 304 lincRNAs and 381 antisense RNAs were identified, of which the expression level was greater than zero only in EAC samples (Fig. 1C). Most of these lncRNAs were upregulated in EAC (Figs. 3C and 3D). These overexpressed lncRNAs were defined as lncRNAs specifically expressed in EAC. Notably, lncRNAs whose expression level was greater than zero only in CSCC samples or in both EAC and CSCC samples were not significantly differentially expressed in any pathological type of cervical cancer. The lncRNA score was calculated to assess its activity in OS, DSS, DFS and PFS analyses and to verify whether EAC-specific lncRNAs serve as a group marker to predict survival and recurrence in patients with cervical cancer. Interestingly, high lncRNA expression was associated with a poor recurrence prognosis (DFS and PFS) rather than shorter survival (OS and DSS) (Fig. 4A). In addition, EAC-specific lncRNA expression may represent an independent prognostic factor for recurrence, and the high expression group had a higher rate of EAC samples in patients with cervical cancer (Figs. 4B and 4C). In summary, we identified lncRNAs specifically expressed in EAC that were associated with the pathological type, and the expression of these lncRNAs was an independent prognostic biomarker of recurrence in patients with cervical cancer.

The immune response is vital for the development and progression of cervical cancer (Cui et al., 2018; Heeren et al., 2015; Punt et al., 2015). Based on accumulating evidence, lncRNAs also serve as vital regulators of gene expression in the immune response (Atianand, Caffrey & Fitzgerald, 2017; Chen, Satpathy & Chang, 2017; Peng et al., 2020; Robinson, Covarrubias & Carpenter, 2020). According to GSEA, negative enrichment of immune cell infiltration signatures, such as T cells and B cells, were observed after the EAC-specific overexpression of lncRNAs (Fig. 5). Furthermore, the correlation analysis showed that overexpression of these lncRNAs was significantly positively correlated with tumor purity and the EAC pathological type and negatively correlated with the CSCC pathological type, immune score, and T cell, B cell, CD8 T cell and NK cell molecular marker activities.

Some lncRNAs specifically expressed in EAC have been reported to have vital functions as oncogenesis and in tumor progression and are associated with unfavorable recurrence outcomes (Ding et al., 2020). Dudek identified that long intergenic nonprotein coding RNA 857 (LINC00857) was upregulated in tumors and was correlated with shorter recurrence-free survival in patients with bladder cancer (Dudek et al., 2018). Li et al. (2017a) identified the upregulation of FOXP4 antisense RNA 1 (FOXP4-AS1) in CRC tissues and cell lines, and its overexpression was positively correlated with advanced pathological stages and a larger tumor size. Li et al. (2017b) identified the upregulation of prostate androgen-regulated transcript 1 (PART1) in tumors, and high PART1 expression indicated shorter DFS in patients with non-small cell lung cancer. Our study also has several limitations. Although we selected some of these lncRNAs to analyze their target genes, we did not explore all these lncRNAs. More and deeper biological mechanisms of these lncRNA markers in cervical cancer need to be further addressed by analyses and experiments.

## Conclusions

In summary, lincRNAs and antisense RNAs were overexpressed in EAC. LncRNAs were upregulated in EAC, and they might represent independent prognostic markers of recurrence in patients with cervical cancer. High expression of these lncRNAs was associated with EAC pathology and a low immune response, thereby resulting in a significantly shorter time to recurrence. Low expression of these lncRNAs correlated with CSCC pathology and an increased immune response, thereby resulting in a significantly longer time to recurrence. Furthermore, most of these lncRNAs have not been reported. These lncRNAs could be considered new targets in the treatment of cervical cancer. These findings may help us obtain deeper insights into potential immunotherapy approaches for cervical cancer.

## Supplemental Information

10.7717/peerj.12116/supp-1 Supplemental Information 1 Raw data for Figs. 1–6.

10.7717/peerj.12116/supp-2 Supplemental Information 2 Analysis of lncRNA’s target based on ENCORI dataset. (A) Barplot showing the number of dysregulated cancer types of each target genes from ENCORI dataset. (B) Network of lncRNAs and their target gene from ENCORI dataset. (C) Barplot showing the—logP of enrichment signature based on these target gene from ENCORI dataset.

10.7717/peerj.12116/supp-3 Supplemental Information 3 The correlations between lncRNAs and Bcell, CD4 T cell and CD8 T cell. (A–B) Dot plots showing the correlations between lncRNAs (A: lincRNAs and B: antisense RNA) and B cell, CD4 T cell and CD8 T cell from TIMER. (C–D) Dot plots showing the correlations between lncRNAs (C: lincRNAs and D: antisense RNA) and B cell, CD4 T cell and CD8 T cell from EPIC. (E–F) Dot plots showing the correlations between lncRNAs (E: lincRNAs and F: antisense RNA) and B cell, CD4 T cell and CD8 T cell from CIBERSORT.

10.7717/peerj.12116/supp-4 Supplemental Information 4 Infiltrating Immune Cells related genes.

10.7717/peerj.12116/supp-5 Supplemental Information 5 The names of the LncRNAs and antisense RNAs.

10.7717/peerj.12116/supp-6 Supplemental Information 6 Clinical case information of patients.

## Additional Information and Declarations

### Data Availability

The following information was supplied regarding data availability: The raw measurements are available in the Supplemental File.
