# Supplementary figures and images for: LncRNAs specifically overexpressed in endocervical adenocarcinoma are associated with an unfavorable recurrence prognosis and the immune response
Source: PeerJ. 2021 Sep 21;9:e12116. doi: 10.7717/peerj.12116 (PMC8462375; doi:10.7717/peerj.12116)

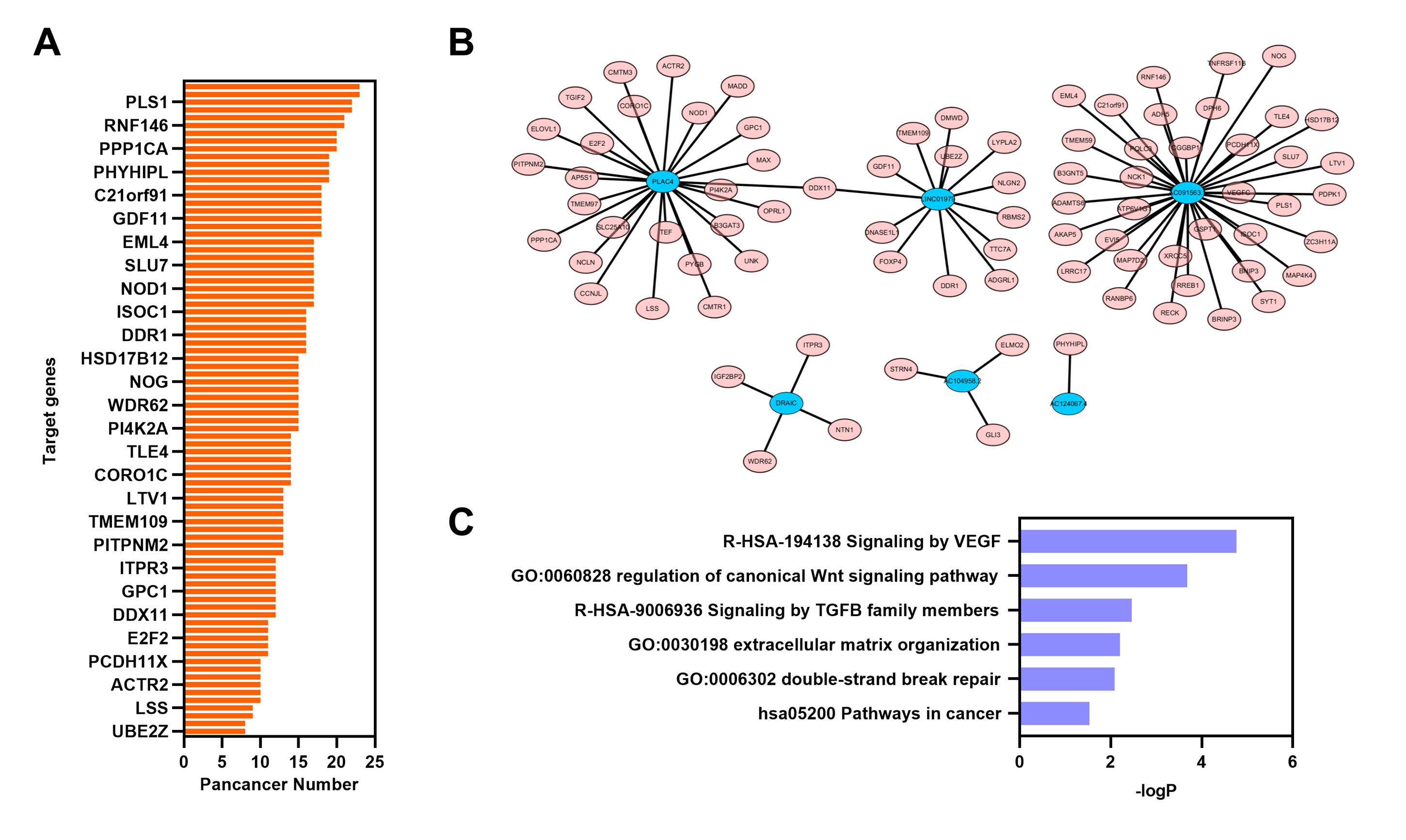

Supplement: Supplemental Information 2 — (A) Barplot showing the number of dysregulated cancer types of each target genes from ENCORI dataset. (B) Network of lncRNAs and their target gene from ENCORI dataset. (C) Barplot showing the—logP of enrichment signature based on these target gene from ENCORI dataset. [file peerj-09-12116-s002.png]

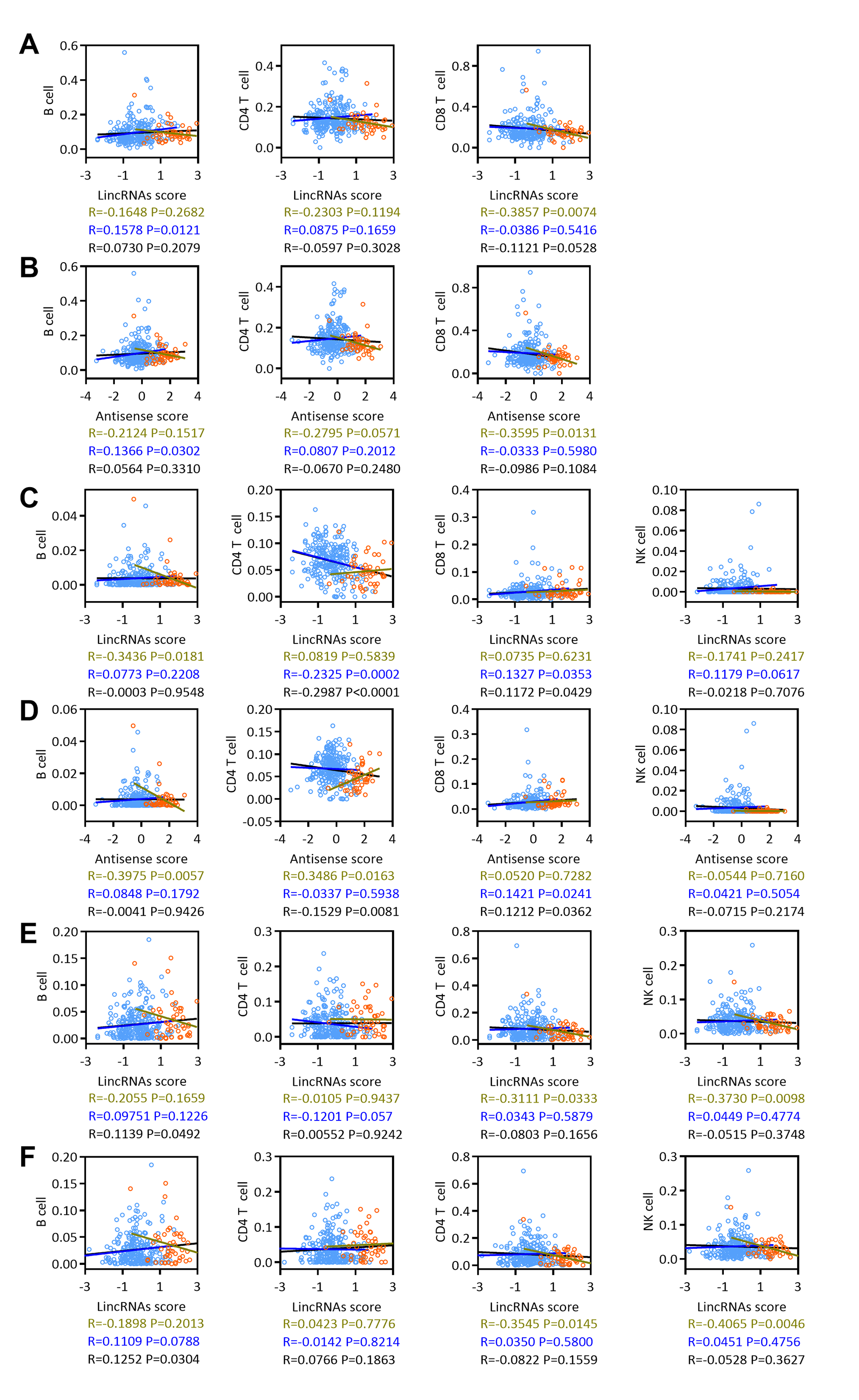

Supplement: Supplemental Information 3 — (A–B) Dot plots showing the correlations between lncRNAs (A: lincRNAs and B: antisense RNA) and B cell, CD4 T cell and CD8 T cell from TIMER. (C–D) Dot plots showing the correlations between lncRNAs (C: lincRNAs and D: antisense RNA) and B cell, CD4 T cell and CD8 T cell from EPIC. (E–F) Dot plots showing the correlations between lncRNAs (E: lincRNAs and F: antisense RNA) and B cell, CD4 T cell and CD8 T cell from CIBERSORT. [file peerj-09-12116-s003.png]
