# Supplementary material for: LncRNAs specifically overexpressed in endocervical adenocarcinoma are associated with an unfavorable recurrence prognosis and the immune response
Source: PeerJ. 2021 Sep 21;9:e12116. doi: 10.7717/peerj.12116 (PMC8462375; doi:10.7717/peerj.12116)
Supplement: Supplemental Information 4 [file peerj-09-12116-s004.docx]

**Table S1** Infiltrating Immune Cells related genes

| Infiltrating Immune Cells | Gene name |
| --- | --- |
| T cell | PRKCQ, CD3D, CD3G, CD28, LCK, TRAT1, BCL11B, CD2, TRBC1, ITM2A, SH2D1A, CD6, CD96, NCALD, GIMAP5, CD3E, SKAP1 |
| B cell | MS4A1, TCL1A, HLA-DOB, PNOC, KIAA0125, CD19, CR2, IGHG1, FCRL2, BLK, COCH, OSBPL10, IGHA1, TNFRSF17, ABCB4, BLNK, GLDC, MEF2C, IGHM, FAM30A, SPIB, BCL11A, GNG7, IGKC, CD72, MICAL3, BACH2, CCR9, QRSL1, DTNB, HLA-DQA1, SCN3A, SLC15A2 |
| CD8 T cell | CD8B, CD8A, PF4, PRR5, SF1, LIME1, DNAJB1, ARHGAP8, GZMM, SLC16A7, SFRS7, APBA2, C4orf15, LEPROTL1, ZFP36L2, GADD45A, MYST3, ZEB1, ZNF609, C12orf47, THUMPD1, VAMP2, ZNF91, ZNF22, TMC6, FLT3LG, CDKN2AIP, TSC22D3, TBCC, RBM3, ABT1, C19orf6, CAMLG, PPP1R2, AES, KLF9, PRF1 |
| NK cell | LOC643313, GAGE2, ZNF747, XCL1, XCL2, AF107846, SLC30A5, MCM3AP, TBXA2R, CDC5L, LOC730096, FUT5, FGF18, MRC2, SPN, PSMD4, PRX, FZR1, ZNF205, AL080130, ZNF528, MAPRE3, BCL2, ARL6IP2, PDLIM4, LDB3, ADARB1, SMEK1, TCTN2, TINAGL1, IGFBP5, ALDH1B1, NCR1 |
