# Supplementary material for: LncRNAs specifically overexpressed in endocervical adenocarcinoma are associated with an unfavorable recurrence prognosis and the immune response
Source: PeerJ. 2021 Sep 21;9:e12116. doi: 10.7717/peerj.12116 (PMC8462375; doi:10.7717/peerj.12116)
Supplement: Supplemental Information 6 [file peerj-09-12116-s006.docx]

**Table S3 Clinical case information of patients**

| Patients ID | 1 | 2 | 3 | 4 | 5 | 6 | 7 | 8 | 9 | 10 | 11 | 12 |
| --- | --- | --- | --- | --- | --- | --- | --- | --- | --- | --- | --- | --- |
| Age(years) | 56 | 47 | 48 | 37 | 67 | 43 | 51 | 43 | 68 | 64 | 46 | 43 |
| Gender | female | female | female | female | female | female | female | female | female | female | female | female |
| Menstrual history | 5-6/28-30 | 5-6/24-26 | 4-6/25-30 | 6-7/32-34 | 5-6/28-30 | 4-6/28-32 | 5-6/29-30 | 5-6/30-31 | 6-7/28-30 | 3-4/28-30 | 5-6/28-30 | 6-7/31-32 |
| Birth history | G3P1 | G1P0 | G5P2 | G3P1 | G4P2 | G3P1 | G2P1 | G4P2 | G4P2 | G5P2 | G5P1 | G3P1 |
| Cervical cancer type | CSCC | CSCC | CSCC | CSCC | CSCC | CSCC | EAC | EAC | EAC | EAC | EAC | EAC |
| Clinical stage | Ⅰ b1 | Ⅰb2 | Ⅰb2 | Ⅰb2 | Ⅱa | Ⅰa2 | Ⅰb2 | Ⅱa | Ⅱa | Ⅰb2 | Ⅰb2 | Ⅰa2 |
| T stage | 1 | 1 | 1 | 1 | 2a | 1 | 1 | 2a | 2a | 1 | 1 | 1 |
| N stage | 0 | 0 | 0 | 1 | 2 | 0 | 2 | 1 | 0 | 1 | 0 | 0 |
| M stage | 0 | 0 | 0 | 0 | 0 | 0 | 0 | 0 | 0 | 0 | 0 | 0 |
